# Survival Outcomes and Treatment Decision by Human Papillomavirus Status Among Patients With Stage IVC Head and Neck Squamous Cell Carcinoma

**DOI:** 10.3389/fonc.2021.668066

**Published:** 2021-05-31

**Authors:** Ping Zhou, Yi-Feng Yu, Chen-Lu Lian, Jun Wang, Ren-Gong Zhuo, San-Gang Wu

**Affiliations:** ^1^ Department of Radiation Oncology, The First Affiliated Hospital of Xiamen University, Xiamen, China; ^2^ Xiamen Key Laboratory of Chiral Drugs, Medical College, Xiamen University, Xiamen, China

**Keywords:** head and neck squamous cell carcinoma, human papillomavirus, prognosis, radiotherapy, decision-making

## Abstract

**Purpose:**

To investigate the influence of human papillomavirus (HPV) status on survival outcomes and treatment decisions for patients with *de novo* stage IV head and neck squamous cell cancers (HNSCC).

**Methods:**

Patients initially diagnosed with *de novo* stage IVC HNSCC between 2010 and 2015 were identified from the Surveillance, Epidemiology, and End Results database. Cox multivariable analyses were performed to determine prognostic factors associated with head and neck cancers specific survival (HNCSS) and overall survival (OS).

**Results:**

We identified 303 patients who received chemotherapy in this study, including 52.5% of them had HPV-positive disease. HPV-positive HNSCC had better HNCSS (P *<* 0.001) and OS (P *<* 0.001) compared to those with HPV-negative disease. The results of Cox multivariable analyses showed that HPV-negative status (P = 0.007), N3 stage (P = 0.004), bone metastases (P < 0.001), and lung metastases (P = 0.003) were associated with worse HNCSS. Similar results were found regarding the OS. The sensitivity analyses indicated that HPV-positive HNSCC patients who were treated with radiotherapy had better survival outcomes. However, no survival benefits were found in those with HPV-positive disease receiving surgery. For HPV-negative patients, no survival benefit was observed among those treated with radiotherapy or surgery.

**Conclusions:**

Approximately half of the stage IVC HNSCC patients are HPV-related. The presence of HPV infection appears to be strongly associated with the survival outcome in patients with *de novo* stage IV HNSCC. Determination of HPV status may help guide clinicians in prognostic assessment and treatment decision-making in this population.

## Introduction

Head and neck cancers (HNC) are the sixth most common solid cancer in the world (1), and the most common pathology type in HNC is squamous cell carcinoma (HNSCC). An estimated over 600,000 new cases of HNC patients and approximately 330,000 HNC-related deaths in 2018 (2). The main reasons for HNC treatment failure are local recurrence and distant metastasis. In addition, approximately 10% of patients have diagnosed with stage IVC disease at the initial diagnosis of HNC (*de novo* stage IVC disease) (3). Patients who presented with stage IVC disease had a dismal prognosis, with a median overall survival (OS) of 10 months (4).

Although tobacco was considered to be an important risk factor for HNSCC, the infection of human papillomavirus (HPV) was identified as a carcinogenic factor for the development of oropharyngeal SCC (OPSCC) (5). A National Cancer Data Base (NCDB) study showed that HPV-positive rates were 62.9, 17.7, 11.0, and 10.6% in patients with oropharynx, hypopharynx, larynx, and oral cavity tumors, respectively (6). Better prognostic effect of HPV-positive OPSCC has been confirmed in recent decades, and its therapeutic effect is significantly better than that of HPV-negative patients (5). However, limited studies have investigated the role of HPV status in patients with *de novo* stage IVC disease (7). Therefore, it is necessary to determine the impact of HPV status on prognosis and treatment decision-making in *de novo* stage IVC HNC patients. In this study, we aimed to evaluate the prevalence, survival outcomes, and treatment decision-making by HPV status for this patient subset using a population-based cohort.

## Material and Methods

### Database and Patient’s Selection Criteria

**Figure 1:**
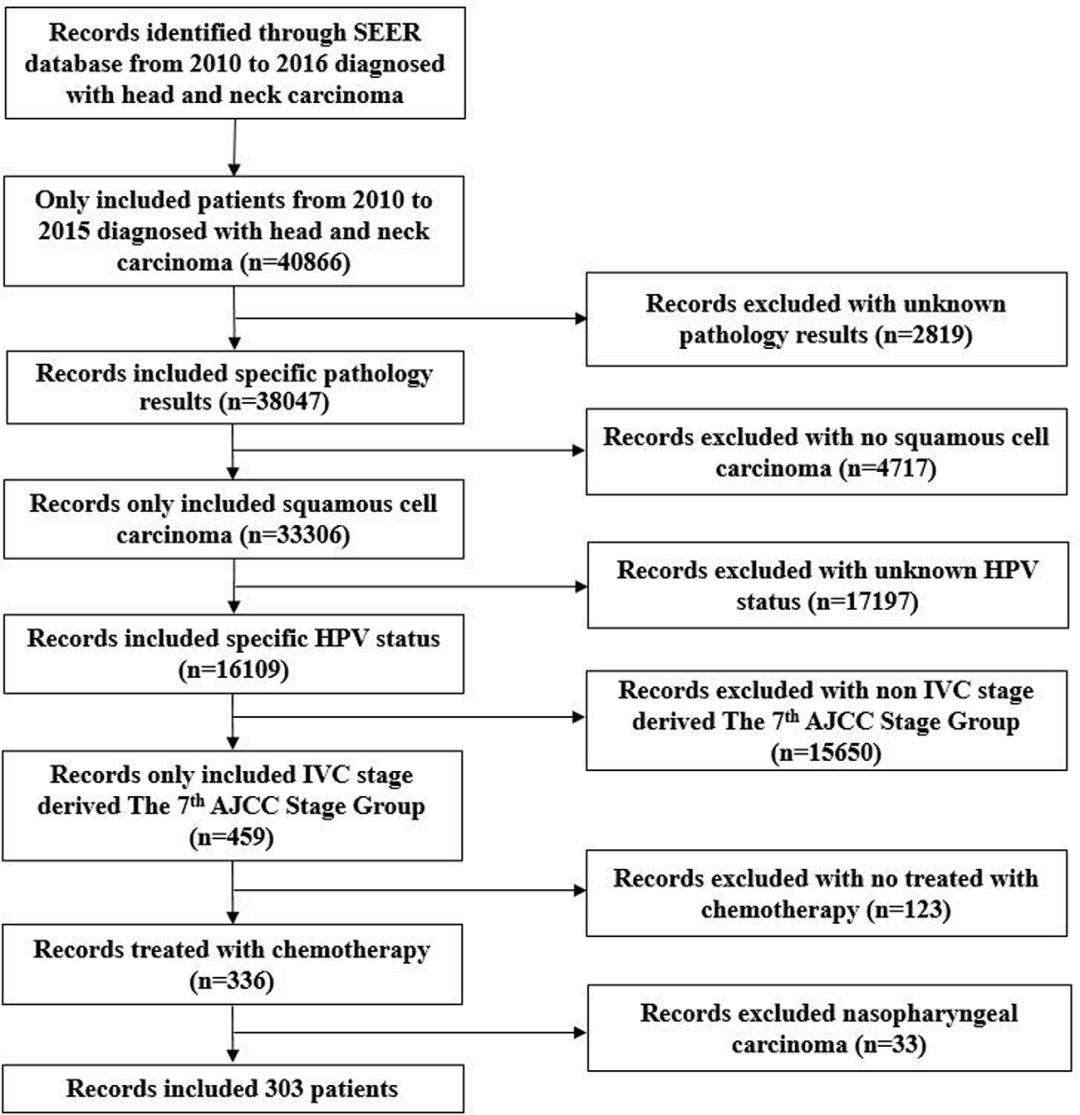
Flow diagram of patient selection and exclusion criteria from the Surveillance, Epidemiology, and End Results database.

In 2018, the SEER program released the database of HPV status regarding the HNC (8). It includes the HPV records and additional treatment fields in 40,866 HNC patients from 2010 to 2016. This database includes patients with the following primary tumor sites: Nasopharynx, Oropharynx, Pharyngeal Tonsil, Pharynx Other, Soft Palate, and Tongue Base, Hypopharynx. The database included HPV status, which were categorized as HPV-positive, HPV-negative, and unknown status. Selection criteria for the current study cohort included: 1) *de novo* stage IVC oropharyngeal SCC or hypopharyngeal SCC; 2) diagnosed between 2010 and 2015; 3) received chemotherapy; 4) known HPV status. Patients with tumors developed in the nasopharynx were excluded. The flow diagram of patient selection is shown in **Figure 1**.

### Data Collection

We examined the demographics, clinicopathological characteristics, and treatment data of patients, including age at diagnosis, gender, race/ethnicity, tumor sites, tumor grade, tumor (T) stage, nodal (N) stage, HPV status, the use of surgery, the use of radiotherapy, and distant metastases sites. The tumor sites of patients were divided into the oropharynx (including pharyngeal tonsil, pharynx other, soft palate, and tongue base) and hypopharynx. The classification of TNM staging was in accordance with the seventh edition of the American Joint Committee on Cancer staging. SEER database only recorded four metastatic sites, including bone, liver, lung, and brain, so we only analyzed the impact of these four metastatic sites on survival. The primary endpoints of this study were head and neck cancers specific survival (HNCSS) and OS. HNCSS was identified as the time from the initial diagnosis of HNSCC to the death of HNC. OS was defined as the time from the initial diagnosis of HNSCC to death from various causes.

### Statistical Analysis

The Chi-square test or Fisher’s exact test were used to compare the difference including demographics, clinicopathological characteristics, and treatment strategy. The Kaplan-Meier methods were used to depict survival curves with log-rank testing. Multivariable Cox regression models were performed to determine whether HPV positive HNSCC was related to better survival outcomes, adjusting for age, gender, race/ethnicity, T stage, N stage, tumor sites, tumor grade, therapeutic strategies, and distant metastases sites. SPSS statistical software (version 25.0, IBM Corporation, Armonk, NY, USA) was used for data analysis. A P < 0.05 was considered to be statistically significant.

## Results

### Patients Clinicopathological Characteristics and Therapeutic Strategies

A total of 303 patients were identified in this study, including 159 patients (52.5%) and 144 (47.5%) patients who had HPV-positive and HPV-negative tumors, respectively. **Table 1** lists the baseline characteristics of patients. Of these patients, the majority were male (n = 253, 83.5%) or Whites (n = 250, 82.5%). There were 182 patients aged younger than 65 years (60.1%). Oropharynx predominated with 89.1% in tumor primary site. In patients with available tumor grade (n = 226), 58.0, 39.4, and 2.7% of them were poorly or undifferentiated, moderately differentiated, and well differentiated, respectively. Most of the patients had nodal positive disease, including 47 (15.8%), 193 (65.0%), and 38 (12.8%) patients who had N1, N2, and N3 diseases, respectively.

**Table 1:** Patient and treatment characteristics by HPV Status.

Variables	n	HPV-negative (%)	HPV-positive (%)	P-value
Age				
<65 years	182 (60.1)	89 (61.8)	93 (58.5)	0.556
≥65 years	121 (39.9)	55 (38.2)	66 (41.5)	
Race/ethnicity				
White	250 (82.5)	111 (77.1)	139 (87.4)	0.052
Black	40 (13.2)	24 (16.7)	16 (10.1)	
Other	13 (4.3)	9 (6.3)	4 (2.5)	
Gender				
Male	253 (83.5)	118 (81.9)	135 (84.9)	0.488
Female	50 (16.5)	26 (18.1)	24 (15.1)	
Primary Site				
Oropharynx	270 (89.1)	118 (81.9)	152 (95.6)	0.000
Hypopharynx	33 (10.9)	26 (18.1)	7 (4.4)	
Grade				
Well differentiated	6 (2.7)	4 (3.5)	2 (1.8)	0.046
Moderately differentiated	89 (39.4)	53 (46.5)	36 (32.1)	
Poorly/undifferentiated	131(58.0)	57 (50.0)	74 (66.1)	
Unknown	77	30	47	
Tumor stage				
T1	32 (12.6)	16 (13.9)	16 (11.6)	0.082
T2	78 (30.8)	26 (22.6)	52 (37.7)	
T3	58 (22.9)	30 (26.1)	28 (20.3)	
T4	85 (33.6)	43 (37.4)	42 (30.4)	
TX	50	29	21	
Nodal stage				
N0	19 (6.4)	11 (7.9)	8 (5.1)	0.001
N1	47 (15.8)	33 (23.7)	14 (8.9)	
N2	193 (65.0)	75 (54.0)	118 (74.7)	
N3	38 (12.8)	20 (14.4)	18 (11.4)	
NX	6	5	1	
Treatment paradigm				
C	82 (27.1)	44 (30.6)	38 (23.9)	0.033
C+R	181 (59.7)	86 (59.6)	95 (59.7)	
C+S	11 (3.6)	7 (4.9)	4 (2.5)	
C+R+S	29 (9.6)	7 (4.9)	22 (13.8)	
Bone metastases				
No	220 (74.3)	103 (73.0)	117 (75.5)	0.632
Yes	76 (25.7)	38 (27.0)	38 (24.5)	
Unknown	7	3	4	
Brain metastases				
No	289 (98.0)	135 (96.4)	154 (99.4)	0.172
Yes	6 (2.0)	5 (3.6)	1 (0.6)	
Unknown	8	4	4	
Liver metastases				
No	254 (86.1)	119 (85.0)	135 (87.1)	0.603
Yes	41 (13.9)	21 (15.0)	20 (12.9)	
Unknown	8	4	4	
Lung metastases				
No	151 (51.0)	68 (47.9)	83 (53.9)	0.302
Yes	145 (49.0)	74 (52.1)	71 (46.1)	
Unknown	7	2	5	

C, Chemotherapy; N, nodal; R, Radiotherapy; S, Surgery; T, tumor; X, unknown.

In patients with available the four mentioned metastatic sites (n = 294), the most common site of distant metastasis was the lung (n = 143, 48.6%), followed by bone (n = 75, 25.5%), liver (n = 41, 13.9%), and brain (n = 6, 2.0%). Of these patients, 59.9% (n = 176), 11.6% (n = 34), and 2.4% (n = 7) of patients metastasized to one site, two, and three sites, respectively. No patients had four metastatic sites.

All patients were treated by chemotherapy, and 72.9% of patients received additional therapies, including radiotherapy (59.7%), surgery (3.6%), and both (9.6%). A total of 40 patients were treated with local surgery. In those with tumors located in the oropharynx, 12 received local tumor excision, 22 treated with pharyngectomy, and 2 patients with unknown surgery procedures. In patients with tumors located in the hypopharynx, three patients received local tumor excision and one patient received pharyngectomy. The demographic factors, clinicopathological characteristics, or treatment factors were not associated with the receipt of surgery.

In general, patients with HPV-related disease were more likely to be OPSCC (P < 0.001), higher tumor grade (P = 0.046), and advanced N stage (P = 0.001). Moreover, HPV-positive patients received more aggressive local treatments including local surgery and radiotherapy compared to those with HPV-negative tumors (13.8 *vs.* 4.9%, P = 0.033). No significant differences were found between patients with HPV-positive and HPV-negative diseases regarding age at diagnosis (P = 0.556), race/ethnicity (P = 0.052), gender (P = 0.488), T stage (P = 0.082), and distant metastatic sites (all P > 0.05).

### Survival Outcomes

**Figure 2:**
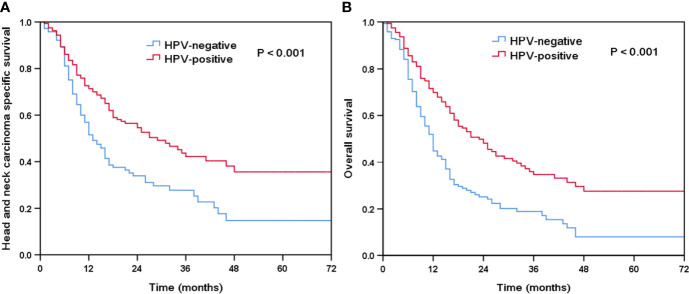
Kaplan-Meier plots of head and neck carcinoma specific survival **(A)** and overall survival **(B)** by HPV status.

The median follow-up time was 15 months (range, 0–78 months). There were 212 deaths observed, of which 173 patients died for HNC-related disease. The Kaplan-Meier survival curves for HPV-positive and HPV-negative tumors are shown in **Figure 2**. Patients with HPV-positive diseases had better survival outcomes compared to those with HPV-negative diseases. The 3-year HNCSS was 42.2 and 27.7% in those with HPV-positive and HPV-negative diseases, respectively (P < 0.001, **Figure 2A**), with a median HNCSS of 29 and 13 months, respectively. Unadjusted median OS was 12 months for HPV-negative patients compared to 23 months for HPV-positive patients (P < 0.001). The 3-year OS was 34.1 and 18.9% in those with HPV-positive and HPV-negative diseases, respectively (P < 0.001, **Figure 2B**).

### Prognostic Analyses

Using multivariate Cox regression analysis, prognostic factors related to HNCSS and OS were investigated (**Table 2**). The results showed that HPV status, bone metastasis, lung metastasis, and advanced nodal stage were the independent prognostic factors associated with HNCSS and OS. HPV-positive patients had better HNCSS (hazard ratio [HR] 0.600, 95% confidence interval [CI] 0.415–0.867, P = 0.007) and OS (HR 0.560, 95% CI 0.401–0.782, P = 0.001) compared to those HPV-negative patients. Compared to patients with HPV-negative disease, those with HPV-positive disease had a 40 and 44% decreased risk of HNC-specific mortality and overall mortality, respectively. However, age at diagnosis, race/ethnicity, gender, tumor sites, grade, T stage, the addition of local treatment to chemotherapy, brain metastatic disease, and liver metastatic disease were not associated with survival outcomes.

**Table 2:** Multivariate Cox proportional hazards models of overall survival and head and neck squamous cell carcinoma specific survival.

Variables	OS		HNCSS	
	HR (95% CI)	P-value	HR (95% CI)	P-value
Age				
<65 years	Ref.		Ref.	
≥65 years	0.803 (0.593–1.088)	0.157	0.728 (0.517–1.024)	0.068
Race				
White	Ref.		Ref.	
Black	0.855 (0.557–1.311)	0.472	0.927 (0.578–1.486)	0.753
Other	0.937 (0.445–1.972)	0.864	1.217 (0.571–2.597)	0.611
Gender				
Male	Ref.		Ref.	
Female	1.051 (0.710–1.555)	0.806	0.916 (0.576–1.458)	0.713
Primary Site				
Oropharynx	Ref.		Ref.	
Hypopharynx	1.156 (0.740–1.806)	0.524	1.116 (0.673–1.851)	0.671
Grade				
Well differentiated	Ref.		Ref.	
Moderately differentiated	0.724 (0.273–1.917)	0.515	0.562 (0.210–1.504)	0.251
Poorly/undifferentiated	0.598 (0.226–1.583)	0.301	0.441 (0.165–1.181)	0.103
Tumor stage				
T1	Ref.		Ref.	
T2	1.215 (0.687–2.150)	0.503	1.200 (0.341–2.273)	0.575
T3	1.486 (0.829–2.663)	0.184	1.365 (0.711–2.660)	0.349
T4	1.635 (0.920–2.809)	0.095	1.511 (0.811–2.815)	0.193
Nodal Stage				
N0	Ref.		Ref.	
N1	1.072 (0.558–2.060)	0.835	1.105 (0.500–2.438)	0.805
N2	1.241 (0.715–2.156)	0.443	1.526 (0.784–2.973)	0.214
N3	2.390 (1.259–4.536)	0.008	3.016 (1.422–6.397)	0.004
Treatment paradigm				
C	Ref.		Ref.	
C+R	0.909 (0.650–1.271)	0.577	0.920 (0.637–1.328)	0.655
C+S	1.861 (0.918–3.776)	0.085	1.392 (0.573–3.385)	0.465
C+R+S	0.710 (0.390–1.292)	0.262	0.698 (0.361–1.348)	0.284
Bone metastases				
No	Ref.		Ref.	
Yes	2.199 (1.563–3.093)	0.000	2.482 (1.719–3.584)	0.000
Brain metastases				
No	Ref.		Ref.	
Yes	0.794 (0.303–2.080)	0.639	0.857 (0.322–2.279)	0.757
Liver metastases				
No	Ref.		Ref.	
Yes	1.241 (0.798–1.930)	0.339	1.380 (0.961–2.213)	0.181
Lung metastases				
No	Ref.		Ref.	
Yes	1.533 (1.115–2.108)	0.008	1.702 (1.201–2.401)	0.003
HPV status				
Negative	Ref.		Ref.	
Positive	0.560 (0.401–0.782)	0.001	0.600 (0.415–0.867)	0.007

C, Chemotherapy; CI, confidence interval; HNCSS, head and neck cancers specific survival; HR, hazard ratio; HPV, human papillomavirus; Ref., reference; OS, overall survival; R, Radiotherapy; S, Surgery.

### Effect of HPV Status on Prognosis After Stratification by Tumor Location

**Figure 3:**
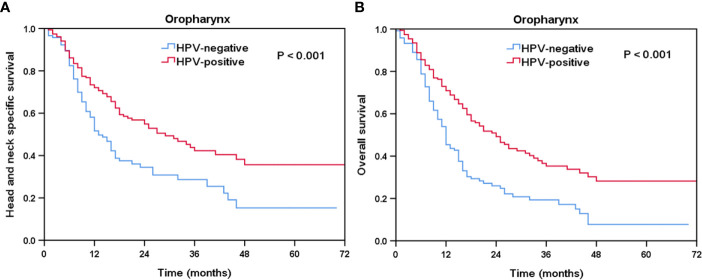
Kaplan-Meier plots of head and neck carcinoma specific survival **(A)** and overall survival **(B)** in oropharyngeal carcinoma by HPV status.

Furthermore, we performed the sensitivity analyses to determine the effect of HPV status on survival outcomes after stratification by tumor location (**Table 3**). The results showed that HPV status was an independent prognostic factor for oropharyngeal cancer, but not for hypopharyngeal cancer. In OPSCC, patients with HPV-positive disease had better HNCSS (HR 0.573, 95% CI 0.416–0.789, P = 0.001) and OS (HR 0.524, 95% CI 0.392–0.701, P < 0.001) compared to those with HPV-negative disease. Comparable survival outcomes were found between HPV-negative and HPV-related tumors in hypopharyngeal cancer. The Kaplan-Meier survival curves for HNCSS and OS by sensitivity analyses are listed in **Figure 3**.

**Table 3:** Sensitivity analyses.

Variables	OS		HNCSS	
	HR (95% CI)	P-value	HR (95% CI)	P-value
Primary site				
Oropharynx				
HPV-negative	Ref.		Ref.	
HPV-positive	0.524 (0.392–0.701)	0	0.573 (0.416–0.789)	0.001
Hypopharynx				
HPV-negative	Ref.		Ref.	
HPV-positive	0.810 (0.304–2.159)	0.601	0.621 (0.180–2.141)	0.451
HPV-positive tumors				
Radiotherapy				
No	Ref.		Ref.	
Yes	0.569 (0.371–0.872)	0.01	0.569 (0.358–0.905)	0.017
Surgery				
No	Ref.		Ref.	
Yes	0.745 (0.422–1.314)	0.309	0.549 (0.274–1.099)	0.091
HPV-negative tumors				
Radiotherapy				
No	Ref.		Ref.	
Yes	0.712 (0.489–1.037)	0.077	0.736 (0.483–1.124)	0.156
Surgery				
No	Ref.		Ref.	
Yes	1.150 (0.631–2.096)	0.648	1.184 (0.613–2.289)	0.615

CI, confidence interval; HNCSS, head and neck cancers specific survival; HR, hazard ratio; HPV, human papillomavirus; Ref., reference; OS, overall survival.

### Effect of Local Treatment on Survival According to HPV Status

**Figure 4:**
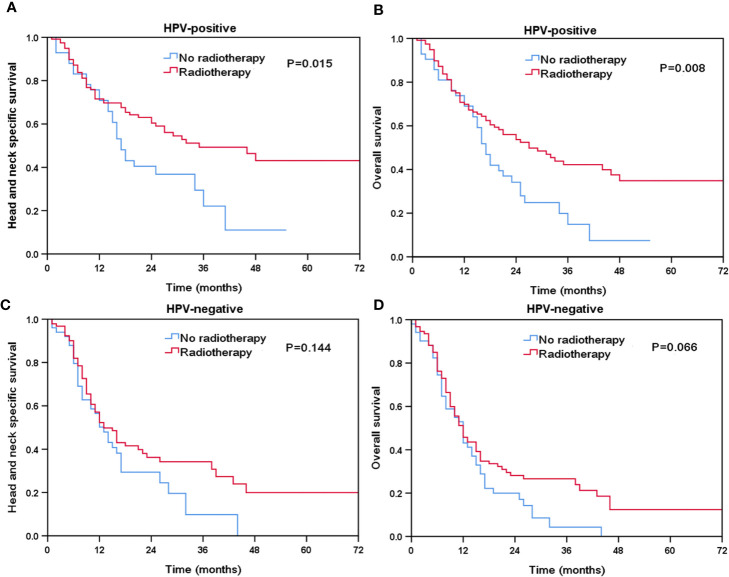
The effect of additional radiotherapy in head and neck cancer-specific survival by HPV status (**A**, HPV-positive; **C**, HPV-negative****) and overall survival (**B**, HPV-positive; **D**, HPV-negative****).

**Figure 5:**
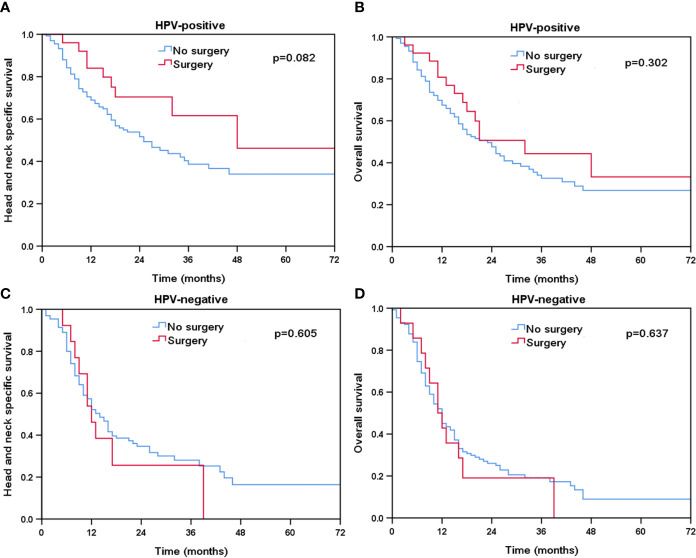
The effect of additional surgery in head and neck cancer-specific survival by HPV status (**A**, HPV-positive; **C**, HPV-negative) and overall survival (**B**, HPV-positive; **D**, HPV-negative).

Finally, we performed sensitivity analyses to determine the effect of local treatment on survival according to HPV status (**Table 3**). For patients with HPV-positive tumors, additional radiotherapy was associated with a better HNCSS (HR 0.569, 95% CI 0.358–0.905, P = 0.017) and OS (HR 0.569, 95% CI 0.371–0.872, P = 0.010) compared to those without receiving radiotherapy. However, comparable HNCSS (HR 0.549, 95% CI 0.274–1.099, P = 0.091) and OS (HR 0.745, 95% CI 0.422–1.314, P = 0.309) were found between surgery and no surgery cohorts. For HPV-negative patients, no survival benefits were found in those treated with radiotherapy or surgery. The Kaplan-Meier survival curves for HNCSS and OS by sensitivity analyses are listed in **Figures 4** and **5**.

## Discussion

In the study, we aimed to explore the effect of HPV status on prognosis and treatment decision-making in patients with *de novo* stage IVC HNSCC. Our study showed that 52.5% of *de novo* stage IVC HNSCC patients were HPV-related, and patients with HPV-positive tumors had significantly better survival outcomes compared to HPV-negative patients, especially for those with OPSCC. Additional radiotherapy improved survival for this patient subset with HPV-positive disease.

The history of HPV in HNC can be traced date back to 1901 (9). In 1983, HPV infection was found in a subtype of OPSCC (10). With the increasing knowledge of HPV in HNC, we could find that different primary tumor sites of HNSCC showed significant differences regarding the HPV status (6, 11). In addition, the incidence of HPV infection also has a significant difference between Westerns and Asians (6, 11–13). A previous study by Chaturvedi et al. included patients from the United States, they found that the incidence of HPV infection increased by 225% from 1988 to 2004 among OPSCC patients (14). A large cohort by Tian et al. from NCDB included 247,040 HNSCC patients, they found that 62.9, 17.7, 11.0, and 10.6% of patients tumor located in the oropharynx, hypopharynx, larynx, and oral cavity were HPV-related (6). A higher rate of HPV infection (70%) was also found in Sweden among those with tonsillar and base of tongue SCC (12). However, in southern China, HPV infection was only detected in 26.4% of HNSCC, and 38.6% of OPSCC patients were HPV-related (11). A study from Japan showed that HPV-positive diseases arising in the oropharynx, oral cavity, nasopharynx, hypopharynx, larynx were 34.4, 0, 2.0, 3.5, and 3.9%, respectively (13). In this study, we only included patients with *de novo* stage IVC HNSCC, and we found that the incidence of HPV-positive OPSCC was higher than the Asians studies (56.3 vs. 34.4–38.6%) and lower than the Western study (56.3 *vs.* 62.9–70.0%) (6, 11–13). The underlying reason was that HPV might affect the staging of patients (15), HPV-positive patients may have a lower risk of distant metastases (16). However, our study indicated there was no significant difference in the incidence of HPV-positive between patients with and without distant metastasis when initially diagnosed. In addition, the patient inclusion criterion and the measuring method of the HPV status probably also take part in this variability. Moreover, tobacco use which was not mentioned in the SEER study may also contribute to the discrepancy in HPV status (17).

There were several distinct clinicopathological features in HPV-related tumors compared to those with HPV-negative tumors. HPV-positive patients were younger and rarely overindulged in alcohol consumption or tobacco use (17). Additionally, HPV-positive tumors were more common in the oropharynx, while smoking-related tumors were more common in the non-oropharyngeal (18). A large cohort study that included 41,950 HNSCC patients collected from NCDB showed that HPV-positive patients were more likely to be younger age, Caucasians, male, and poor tumor differentiation (19). A study showed that advanced stage HPV-positive OPSCC patients had an earlier T stage than HPV negative patients (20). HPV-related OPSCC patients often presented with advanced N stage (15). In our study, we found a higher proportion of HPV-positive *de novo* stage IVC HNSCC patients were poor differentiation, which was similar to those patients with non-metastatic HNSCC (17–19). In addition, we also found that HPV-positive tumors were more likely to be diagnosed with the advanced nodal stage (N2 and N3), which was similar to the previous studies in patients with non-metastatic disease (15).

Better prognostic effect of HPV-positive OPSCC has been confirmed in a recent decade (5, 19, 20). However, the prognostic value of HPV status in other sites of HNC remains unclear. Ni G et al. found that HPV-related HNSCC was a remarkably longer stage-specific survival time than patients without HPV infection (11). Burr et al. identified 708 stage IVC non-OPSCC patients selected from the NCDB found that HPV-positive patients in oral cavity disease had improved prognosis, but no survival benefit was found in HPV-positive tumors located in the hypopharynx and larynx (7). Similar results were found in stage III-IV laryngeal/hypopharyngeal cancers who receiving primary radiotherapy (21). A study including 218 advanced HNSCC patients found that there was no notable relation between response to therapy and survival outcomes regarding the HPV status (22). Most of the current researches focus on nonmetastatic HNSCC patients. However, in our cohort, we suggested that HPV status was also an important prognostic factor for *de novo* stage IVC oropharyngeal carcinoma patients. In the new AJCC staging (23), HPV status was added in oropharyngeal cancer staging, but it did not stratify the risk of IVC patients. Therefore, the HPV status might be also integrated into the staging of *de novo* stage IVC oropharyngeal carcinoma patients in the future edition of the AJCC staging system.

Regarding distant metastases, Bollig et al. also found that the lung and bone were the most common metastatic sites of HNC, and bone metastasis was the independent prognostic factor for worse outcomes (24). Similar results were found in our study. An inferior prognosis in patients with bone metastasis was also found in the study by Schulz et al. (25). The reasons for inferior outcomes for patients with bone metastasis remain unclear, potential confounding factors that may have contributed to this phenomenon. In addition, several studies have shown that bone metastasis often shows a rapidly deteriorating clinical course and extremely poor survival outcomes, which is related to the fact that bone metastasis is easy to combine with bone marrow metastasis and cause serious hematologic abnormalities such as disseminated intravascular coagulation (26–29).

With increasing trends of HPV testing in patients with HNSCC, current studies focused on the adjustment of HPV-related treatment strategies to reduce treatment-related toxicity. There were several treatment strategies including a reduction in the dose of radiotherapy, the replacement of cisplatin in concurrent chemoradiotherapy, less invasive surgical options, and immunotherapy (29–32). However, it raised the question that whether the HPV status should be considered in decision-making in patients with stage IVC disease. To our knowledge, there was no research to explore the treatment decision-making for patients with stage IVC according to HPV status. According to NCCN guidelines, systemic chemotherapy and supportive care was the primary treatment mode, surgical or radiation therapy could be considered for selected metastatic patients (33). In this study, we further analyzed the impact of HPV status on survival in different local treatment strategies. Our results showed that for *de novo* stage IVC HNSCC patients, after treated by chemotherapy, the addition of radiotherapy had a better outcome in HPV-positive patients but not in HPV-negative patients. However, the addition of surgery didn’t bring survival benefits in HPV-positive or negative patients. It is a hypothesis that HPV-positive patients receive more aggressive local treatment, which may be related to a higher radiation sensitivity and a better prognosis in HPV related-disease.

Higher survival outcomes in HPV-positive oropharyngeal cancer patients may partly because of the greater locoregional control induced by radiation with higher inherent radiosensitivity or better radiosensitivity induced by cisplatin (5). Furthermore, lower TP53 mutations, a decrease of cell repairability, lower expression of SMG-1 protein, dysregulation of the cell cycle, and response for DNA damage were associated with radiosensitivity for HPV positive patients (34–37). Regarding hypopharyngeal carcinoma, it seems that HPV status was not associated with the prognosis in this study. It was still controversial whether there was a prognostic value in non-metastatic hypopharyngeal carcinoma. Previous studies regarding the role of HPV status on the prognosis of hypopharyngeal carcinoma including the small size of patients (21, 22). Therefore, it is necessary to further study the value of HPV status in stage IVC patients with hypopharyngeal cancer.

Our study provides important data for the discrepancy with different HPV status in the patients with *de novo* stage IVC HNSCC. However, there were several limitations in the current study. First, the findings came from a retrospective observational study with a relatively small sample size. Second, the non-randomized allocation of local treatment may raise selection bias. Third, the methods of HPV testing may be heterogeneous. In addition, the additional abuse of tobacco and alcohol was also not recorded in the SEER database, which might influence the results. Finally, SEER also lacks information regarding the chemotherapy regimen, molecular targeting therapy, sequence of systemic and local treatments, the number of chemotherapy cycles, response of treatment, tobacco/alcohol addiction, and patient’s performance status. Despite these limitations, we believe that our findings are enlightening enough to further study the impact of HPV status on prognosis and decision-making. The primary strength of our study was that we used a population-based cohort to investigate explore the effect of HPV status on prognosis and treatment decision making in patients with *de novo* stage IVC HNSCC, which may help clinicians to develop a better risk stratification and precise treatment decision-making for this patient subset.

## Conclusion

In conclusion, our study suggests that approximately half of the stage IVC HNSCC patients are HPV-related. The presence of HPV infection appears to be strongly associated with the survival outcome in patients with *de novo* stage IV HNSCC. Determination of HPV status may help guide clinicians in prognostic assessment and treatment decision-making in this population. Future studies on HPV testing in patients with *de novo* stage IV HNSCC are needed to determine the risk stratification and treatment recommendations in this population.

## Data Availability Statement

Publicly available datasets were analyzed in this study. This data can be found here: www.seer.cancer.gov.

## Ethics Statement

Ethical review and approval was not required for the study on human participants in accordance with the local legislation and institutional requirements. Written informed consent for participation was not required for this study in accordance with the national legislation and the institutional requirements.

## Author Contributions

S-GW, PZ, Y-FY, JW, and C-LL are lead authors who participated in data collection, manuscript drafting, tables/figures creation, and manuscript revision. S-GW aided in data collection. PZ and Y-FY are senior authors who aided in drafting the manuscript and manuscript revision. R-GZ and S-GW are the corresponding authors who initially developed the concept and drafted and revised the manuscript. All authors contributed to the article and approved the submitted version.

## Conflict of Interest

The authors declare that the research was conducted in the absence of any commercial or financial relationships that could be construed as a potential conflict of interest.

## References

[1] Ferlay J Soerjomataram I Dikshit R Eser S Mathers C Rebelo M. Cancer Incidence and Mortality Worldwide: Sources, Methods and Major Patterns in GLOBOCAN 2012. Int J Cancer (2015) 136(5):E359–86. 10.1002/ijc.29210 25220842

[2] Bray F Ferlay J Soerjomataram I Siegel RL Torre LA Jemal A. Global Cancer Statistics 2018: GLOBOCAN Estimates of Incidence and Mortality Worldwide for 36 Cancers in 185 Countries. CA Cancer J Clin (2018) 68(6):394–424. 10.3322/caac.21492 30207593

[3] Garavello W Ciardo A Spreafico R Gaini RM. Risk Factors for Distant Metastases in Head and Neck Squamous Cell Carcinoma. Arch Otolaryngol Head Neck Surg (2006) 132(7):762–6. 10.1001/archotol.132.7.762 16847186

[4] Vermorken JB Mesia R Rivera F Remenar E Kawecki A Rottey S. Platinum-Based Chemotherapy Plus Cetuximab in Head and Neck Cancer. N Engl J Med (2008) 359(11):1116–27. 10.1056/NEJMoa0802656 18784101

[5] Ang KK Harris J Wheeler R Weber R Rosenthal DI Nguyen-Tân PF. Human Papillomavirus and Survival of Patients With Oropharyngeal Cancer. N Engl J Med (2010) 363(1):24–35. 10.1056/NEJMoa0912217 20530316 PMC2943767

[6] Tian S Switchenko JM Jhaveri J Cassidy RJ Ferris MJ Press RH. Survival Outcomes by High-Risk Human Papillomavirus Status in Nonoropharyngeal Head and Neck Squamous Cell Carcinomas: A Propensity-Scored Analysis of the National Cancer Data Base. Cancer (2019) 125(16):2782–93. 10.1002/cncr.32115 PMC6663628 31012957

[7] Burr AR Harari PM Ko HC Chen S Yu M Baschnagel AM. HPV Impacts Survival of Stage IVC Non-Oropharyngeal HNSCC Cancer Patients. Otorhinolaryngol Head Neck Surg (2018) 3(1):10.15761/OHNS.1000160. 10.15761/OHNS.1000160 PMC6157736 30271885

[8] Surveillance, Epidemiology, and End Results (SEER) Program. SEER*Stat Database: Incidence - SEER 18 Regs Custom Data Head and Neck (select schemas with HPV recode and additional treatment fields), Nov 2018 Sub (2010-2016) - Linked To County Attributes - Total U.S., 1969-2017 Counties. Bethesda, Maryland: National Cancer Institute, DCCPS, Surveillance Research Program (2019). Available at: www.seer.cancer.gov. released April 2019, based on the November 2018 submission.

[9] Syrjänen S Rautava J Syrjänen K. HPV in Head and Neck Cancer-30 Years of History. Recent Results Cancer Res (2017) 206:3–25. 10.1007/978-3-319-43580-0_1 27699526

[10] Syrjänen KJ Pyrhönen S Syrjänen SM Lamberg MA. Immunohistochemical Demonstration of Human Papilloma Virus (HPV) Antigens in Oral Squamous Cell Lesions. Br J Oral Surg (1983) 21(2):147–53. 10.1016/0007-117x(83)90060-4 6307342

[11] Ni G Huang K Luan Y Cao Z Chen S Ma B. Human Papillomavirus Infection Among Head and Neck Squamous Cell Carcinomas in Southern China. PloS One (2019) 14(9):e0221045. 10.1371/journal.pone.0221045 31545798 PMC6756512

[12] Haeggblom L Attoff T Yu J Holzhauser S Vlastos A Mirzae L. Changes in Incidence and Prevalence of Human Papillomavirus in Tonsillar and Base of Tongue Cancer During 2000-2016 in the Stockholm Region and Sweden. Head Neck (2019) 41(6):1583–90. 10.1002/hed.25585 30584688

[13] Maruyama H Yasui T Ishikawa-Fujiwara T Morii E Yamamoto Y Yoshii T. Human Papillomavirus and P53 Mutations in Head and Neck Squamous Cell Carcinoma Among Japanese Population. Cancer Sci (2014) 105(4):409–17. 10.1111/cas.12369 PMC4317800 24521534

[14] Chaturvedi AK Engels EA Pfeiffer RM Hernandez BY Xiao W Kim E. Human Papillomavirus and Rising Oropharyngeal Cancer Incidence in the United States. J Clin Oncol (2011) 29(32):4294–301. 10.1200/JCO.2011.36.4596 PMC3221528 21969503

[15] Wittekindt C Klussmann JP. Tumor Staging and HPV-Related Oropharyngeal Cancer. Recent Results Cancer Res (2017) 206:123–33. 10.1007/978-3-319-43580-0_9 27699534

[16] Grønhøj C Jakobsen KK Jensen DH Rasmussen J Andersen E Friborg J. Pattern of and Survival Following Loco-Regional and Distant Recurrence in Patients With HPV+ and HPV- Oropharyngeal Squamous Cell Carcinoma: A Population-Based Study. Oral Oncol (2018) 83:127–33. 10.1016/j.oraloncology.2018.06.012 30098768

[17] O’Sullivan B Huang SH Siu LL Waldron J Zhao H Perez-Ordonez B. Deintensification Candidate Subgroups in Human Papillomavirus-Related Oropharyngeal Cancer According to Minimal Risk of Distant Metastasis. J Clin Oncol (2013) 31(5):543–50. 10.1200/JCO.2012.44.0164 23295795

[18] Sturgis EM Cinciripini PM. Trends in Head and Neck Cancer Incidence in Relation to Smoking Prevalence: An Emerging Epidemic of Human Papillomavirus-Associated Cancers? Cancer (2007) 110(7):1429–35. 10.1002/cncr.22963 17724670

[19] Li H Torabi SJ Yarbrough WG Mehra S Osborn HA Judson B. Association of Human Papillomavirus Status At Head and Neck Carcinoma Subsites With Overall Survival. JAMA Otolaryngol Head Neck Surg (2018) 144(6):519–25. 10.1001/jamaoto.2018.0395 PMC6583856 29801040

[20] Ward MJ Mellows T Harris S Webb A Patel NN Cox HJ. Staging and Treatment of Oropharyngeal Cancer in the Human Papillomavirus Era. Head Neck (2015) 37(7):1002–13. 10.1002/hed.23697 24753272

[21] Hughes RT Beuerlein WJ O’Neill SS Porosnicu M Lycan TW Waltonen JD. Human Papillomavirus-Associated Squamous Cell Carcinoma of the Larynx or Hypopharynx: Clinical Outcomes and Implications for Laryngeal Preservation. Oral Oncol (2019) 98:20–7. 10.1016/j.oraloncology.2019.09.008 PMC7457446 31536842

[22] Descamps G Karaca Y Lechien JR Kindt N Decaestecker C Remmelink M. Classical Risk Factors, But Not HPV Status, Predict Survival After Chemoradiotherapy in Advanced Head and Neck Cancer Patients. J Cancer Res Clin Oncol (2016) 142(10):2185–96. 10.1007/s00432-016-2203-7 PMC5018052 27370781

[23] Amin MB Edge S Greene F Byrd DR Brookland RK Washington MK. Ajcc Cancer Staging Manual. 8th ed. New York, NY: Springer International Publishing (2016).

[24] Bollig CA Newberry CI Galloway TLI Zitsch RP Hanly EK Zhu VL. Prognostic Impact of Metastatic Site and Pattern in Patients With Metastatic Head and Neck Cancer. Laryngoscope (2020) 131(6):E1838–46. 10.1002/lary.29208 PMC8896291 33098338

[25] Schulz D Wirth M Piontek G Knopf A Straube C Pigorsch S. Improved Overall Survival in Head and Neck Cancer Patients After Specific Therapy of Distant Metastases. Eur Arch Otorhinolaryngol (2018) 275(5):1239–47. 10.1007/s00405-018-4920-9 29520497

[26] Kim YJ Kim SH Kim JW Lee JO Kim JH Bang SM. Gastric Cancer With Initial Bone Metastasis: A Distinct Group of Diseases With Poor Prognosis. Eur J Cancer (2014) 50(16):2810–21. 10.1016/j.ejca.2014.08.003 25201165

[27] Rhee J Han SW Oh DY Im SA Kim TY Bang YJ. Clinicopathologic Features and Clinical Outcomes of Gastric Cancer That Initially Presents With Disseminated Intravascular Coagulation: A Retrospective Study. J Gastroenterol Hepatol (2010) 25(9):1537–42. 10.1111/j.1440-1746.2010.06289.x 20796152

[28] Kwon JY Yun J Kim HJ Kim KH Kim SH Lee SC. Clinical Outcome of Gastric Cancer Patients With Bone Marrow Metastases. Cancer Res Treat (2011) 43(4):244–9. 10.4143/crt.2011.43.4.244 PMC3253867 22247710

[29] Wierzbicka M Szyfter K Milecki P Składowski K Ramlau R. The Rationale for HPV-Related Oropharyngeal Cancer De-Escalation Treatment Strategies. Contemp Oncol (Pozn) (2015) 19(4):313–22. 10.5114/wo.2015.54389 PMC4631307 26557780

[30] Stock GT Bonadio RRCC de Castro G. De-Escalation Treatment of Human Papillomavirus-Positive Oropharyngeal Squamous Cell Carcinoma: An Evidence-Based Review for the Locally Advanced Disease. Curr Opin Oncol (2018) 30(3):146–51. 10.1097/CCO.0000000000000441 29474271

[31] Chen AM Felix C Wang PC Hsu S Basehart V Garst J. Reduced-Dose Radiotherapy for Human Papillomavirus-Associated Squamous-Cell Carcinoma of the Oropharynx: A Single-Arm, Phase 2 Study. Lancet Oncol (2017) 18(6):803–11. 10.1016/S1470-2045(17)30246-2 PMC6488353 28434660

[32] Anderson CM Kimple RJ Lin A Karam SD Margalit DN Chua MLK. De-Escalation Strategies in HPV-Associated Oropharynx Cancer-Are We Putting the Cart Before the Horse? Int J Radiat Oncol Biol Phys (2019) 104(4):705–9. 10.1016/j.ijrobp.2019.02.054 PMC7194352 31204653

[33] National Comprehensive Cancer Network. NCCN Clinical Practice Guidelines in Oncology: Head and Neck Cancer. V. 1. 2021. Available at: https://www.nccn.org/professionals/physician_gls/pdf/head_and_neck (Accessed Access Jan 15, 2021).

[34] Perrone F Suardi S Pastore E Casieri P Orsenigo M Caramuta S. Molecular and Cytogenetic Subgroups of Oropharyngeal Squamous Cell Carcinoma. Clin Cancer Res (2006) 12(22):6643–51. 10.1158/1078-0432.CCR-06-1759 17121883

[35] Nickson CM Moori P Carter RJ Rubbi CP Parsons JL. Misregulation of DNA Damage Repair Pathways in HPV-Positive Head and Neck Squamous Cell Carcinoma Contributes to Cellular Radiosensitivity. Oncotarget (2017) 8(18):29963–75. 10.18632/oncotarget.16265 PMC5444717 28415784

[36] Gubanova E Brown B Ivanov SV Helleday T Mills GB Yarbrough WG. Downregulation of SMG-1 in HPV-Positive Head and Neck Squamous Cell Carcinoma Due to Promoter Hypermethylation Correlates With Improved Survival. Clin Cancer Res (2012) 18(5):1257–67. 10.1158/1078-0432.CCR-11-2058 PMC4010255 22247495

[37] Stephen JK Divine G Chen KM Chitale D Havard S Worsham MJ. Significance of P16 in Site-Specific HPV Positive and HPV Negative Head and Neck Squamous Cell Carcinoma. Cancer Clin Oncol (2013) 2(1):51–61. 10.5539/cco.v2n1p51 23935769 PMC3736998

